# Association between serum alanine and aspartate aminotransferase and blood pressure: a cross-sectional study of Chinese freshmen

**DOI:** 10.1186/s12872-021-02282-1

**Published:** 2021-10-01

**Authors:** Lijun Zhu, Zhengmei Fang, Yuelong Jin, Weiwei Chang, Mengyun Huang, Lianping He, Yan Chen, Yingshui Yao

**Affiliations:** 1 Department of Epidemiology and Biostatistics, School of Public Health, Wannan Medical College, No. 22, Wenchang Road, Wuhu, 241002 Anhui China; 2 Institute of Chronic Disease Prevention and Control, Wannan Medical College, No. 22, Wenchang Road, Wuhu, 241002 Anhui China; 3 School of Medicine, Taizhou University, Taizhou, 318000 China; 4 Department of Medicine, Anhui College of Traditional Chinese Medicine, No.18, Wuxia Shanxi Road, Wuhu, 241002 Anhui China

**Keywords:** Alanine aminotransferase, Aspartate aminotransferase, Freshmen, Hypertension, Association, Cross-sectional

## Abstract

**Background:**

High blood pressure is a well-recognized risk factor for cardiovascular events, and the incidence of hypertension is increasing among young people. This study investigated the relationship between ALT and AST levels and hypertension among freshmen in China.

**Methods:**

This cross-sectional study was conducted in the Anhui Province from September to November 2018. A total of 3114 freshmen underwent a physical examination including testing of biochemical indicators and a standardized questionnaire.

**Results:**

The overall prevalence of elevated ALT and AST were 6.8% and 2.3% among freshmen. The mean ALT and AST levels were higher in males (22.59 ± 21.98 vs.12.62 ± 10.30 U/L; 23.55 ± 12.24 vs. 20.02 ± 5.75 U/L, respectively). The prevalence of hypertension was significantly higher in men (16.1%) than in women (1.9%). The mean values of BMI, SBP, DBP, TC, TG, and LDL-C were found to be increased with elevated levels of serum ALT and AST in the quartiles (*P* for trend < 0.05). After adjusting for covariates, the risk of hypertension was significantly higher in the highest ALT quartile than in the lowest quartile (OR (95% CI) of 1.681 (1.028, 2.751) in males; 2.802 (1.102, 7.124) in females). A strong linear relationship was found between serum ALT levels and the odds of hypertension after adjustment for potential confounders only in total population and females (*P* for trend < 0.05).

**Conclusions:**

These findings suggest that ALT level is significantly associated with hypertension both in male and female freshmen.

## Background

Cardiovascular diseases are responsible for more than 18 million deaths each year, accounting for approximately one-third of all global deaths [1]. High blood pressure is a well-recognized risk factor for cardiovascular events. Although hypertension is traditionally more prevalent in older people, recent epidemiological studies have shown an increased prevalence of risk factors in the young, including obesity, diabetes mellitus, and renal disease, and the incidence of hypertension is progressively rising among the young [2]. Moreover, the younger the onset of hypertension, the higher the risk of cardiovascular disease and all-cause mortality [3]. Recently, some epidemiological studies suggest liver enzymes such as alanine and aspartate aminotransferase (ALT, AST) are associated with the presence of hypertension [4, 5]. ALT and AST are sensitive indicators of liver cell injury and have been used to identify patients with liver disease. A case–control study in China has showing ALT is a potential indicator for patients with hypertension in senior adults [6]. A 12-year Korean Cohort Study reported that progression to hypertension was associated with frequent drinking and high ALT levels among men [7]. Moreover, previous work from the Framingham Heart Study found that increased ALT levels were associated with elevated blood pressure, and higher AST levels were correlated with higher DBP [8]. Inconsistent with previous studies, Gupta et al. [9] reported no significant relationship in the ALT and AST levels of normal, prehypertensive, and hypertensive individuals. However, most of the previous studies assessed the relationship that included only in adults and their findings were inconsistent. There are scarce earlier studies of this association among young people.

Freshmen generally experience substantial changes including the transition from high school to university, changes in living environment, lifestyle, and life content. A survey in Lebanon found that the students gained approximately 1.90 kg when transitioning from high school to university and significant increases in waist circumference, blood glucose, and triglyceride levels were detected [10]. An entry health examination of college students in North Taiwan shows that the prevalence of abnormal liver function tests was 6.2% among freshmen [11]. A meta-analysis on the prevalence of hypertension among college students in China shows that 3.62% of college students suffer from hypertension [12]. Another study of university students aged 15–19 in Nigeria showed that the prevalence of hypertension was 25.1% [13]. The epidemiological data concerning the association between elevated ALT and AST and hypertension in the Chinese freshmen are not available yet. In view of the relationship between liver enzymes and blood pressure in adults, the study of liver enzymes in adolescents may have guiding significance for mitigating the risk of hypertension among adolescents.

This study aimed to investigate whether there is a relationship between ALT and AST and the occurrence of hypertension among freshmen in China.

## Methods

### Study population

A population-based cross-sectional survey was conducted at a medical college in Anhui, China between September and November 2018. A total of 3450 freshmen were investigated. Another 101 individuals with missing height or weight and 235 individuals with missed questionnaires and associated information were excluded. Ultimately, 3114 freshmen were included in the final analysis. The study was approved by the Ethics Committee of Wannan Medical College, Yijishan Hospital (Approval No. 32 (2018)). Written informed consent was obtained from all participants or, if subjects are under 18, from a parent and/or legal guardian.

### Data collection and measurements

Each participant completed a standard questionnaire and underwent a detailed physical examination by a trained doctor or nurse. Physical examination included the following procedures. Blood pressure was measured twice with an interval of no less than 2 min with a mercury sphygmomanometer (Omron U30, China), with the participants in a sitting position and after at least 5 min of rest. The average value of the two measurements was taken as the final BP. The height and weight of the subjects were measured using an ultrasound height and weight measuring instrument (Shengyuan, China), with the participants in light clothing and without shoes, in an upright position. Waist circumference was measured 1 cm above the umbilicus in a horizontal plane. Body mass index (BMI) was calculated by dividing weight (kg) by height (m) squared (kg/m^2^).

Venous blood samples were collected in the morning after at least 10 h of overnight fasting. The serum samples were separated for biochemical assays without freezing. Biochemical variables, including serum ALT, AST, triglyceride (TG), total cholesterol (TC), high-density lipoprotein cholesterol (HDL-C), low-density lipoprotein cholesterol (LDL-C), and glucose (GLU) were measured using an auto-analyzer.

### Definitions

Hypertension in Chinese adult was defined according to the “2018 Chinese guidelines for the management of hypertension [14]” as systolic blood pressure (SBP) ≥ 140 mm Hg and /or diastolic blood pressure (DBP) ≥ 90 mm Hg, and/or use of antihypertensive medications. For adolescents ≥ 13 years of age, “hypertension” was defined as SBP ≥ 130 mmHg and/or DBP ≥ 80 mmHg [15]. Obesity was diagnosed according to the “Screening for Overweight and Obesity Among School-age Children and Adolescents” criteria: BMI ≥ 27.1, BMI ≥ 27.6, BMI ≥ 28.0 were the obesity criteria for adolescents aged 16, 17, 18 years and older, respectively. Criteria for abnormal liver enzymes for adolescents < 18 years of age were ALT > 30 U/L for boys and > 21 U/L for girls, AST > 38 U/L for boys and > 33 U/L for girls [16]. The upper limit of normal serum ALT level and AST levels both were set at 40 U/L in adults, and an elevated ALT level was defined as > 40 U/L, and elevated AST was defined as > 40 U/L [17]. Serum ALT levels were categorized by quartiles as ≤ 9, 10–12, 13–18, and ≥ 19 U/L. Serum AST levels were categorized by quartiles as ≤ 17, 18–20, 21–23, and ≥ 24 U/L.

### Statistical analysis

We calculated the mean ± standard deviation (SD) for continuous data and frequency (percentage) for categorical variables. The independent-samples t-test was performed to compare the differences in continuous variables. The categorical variable distributions were compared using Chi-square tests. Pearson correlation was used to evaluate the relationship between ALT and AST levels and other variables. Multivariate logistic regression models adjusted for age and sex (Model 1) or age, sex, BMI, smoking, and drinking (Model 2) or Model 2 plus, TC, TG, HDL-C, LDL-C, and GLU were used to estimate the odds ratios (ORs) of the associations between ALT and AST and hypertension. All statistical analyses were conducted using SPSS software (version 18.0; SPSS Inc., Chicago, IL, USA). Statistical significance was defined as a two-tailed *P* < 0.05.

## Results

### Baseline characteristics of this study

The baseline characteristics of the study population according to sex are presented in Table 1. A total of 3114 freshmen (1264 men and 1850 women) participated in the study. The mean age was 18.52 ± 1.01 years (range 15—27 years). The overall prevalence of elevated ALT and AST were 6.8% (n = 211), 2.3% (n = 71) among freshmen. The mean ALT and AST levels were higher in males (22.59 ± 21.98 vs.12.62 ± 10.30 U/L; 23.55 ± 12.24 vs. 20.02 ± 5.75 U/L, respectively). Male freshmen had a significantly higher prevalence of hypertension (16.1%) than female freshmen (1.9%). Additionally, male participants had higher levels of BMI, SBP, DBP, TG, and LDL-C, but lower GLU, TC, and HDL-C levels than the females (*P* < 0.05).

**Table 1:** Characteristics of the study participants

Characteristic	Total (n = 3114)	Males (n = 1264)	Females (n = 1850)	*t*/*χ*^2^	*P*
Age (year)	18.52 ± 1.01	18.57 ± 1.01	18.48 ± 1.01	2.417	0.016
BMI (kg/m^2^)	22.33 ± 3.53	23.11 ± 3.97	21.8 ± 3.09	9.868	0.000
SBP (mmHg)	114.05 ± 13.73	121.71 ± 13.57	108.81 ± 11.14	27.966	0.000
DBP (mmHg)	70.94 ± 10.56	73.21 ± 11.04	69.38 ± 9.92	9.904	0.000
GLU (mmol/L)	4.57 ± 0.4	4.51 ± 0.38	4.6 ± 0.41	− 6.271	0.000
TC (mmol/L)	4.05 ± 0.68	4.01 ± 0.74	4.08 ± 0.63	− 2.95	0.001
TG (mmol/L)	0.83 ± 0.4	0.87 ± 0.47	0.81 ± 0.35	3.826	0.000
HDL-C (mmol/L)	1.42 ± 0.28	1.31 ± 0.25	1.49 ± 0.27	− 18.915	0.000
LDL-C (mmol/L)	2.09 ± 0.56	2.15 ± 0.63	2.05 ± 0.5	4.846	0.000
ALT (U/L)	16.67 ± 16.82	22.59 ± 21.98	12.62 ± 10.30	15.035	0.000
AST (U/L)	21.45 ± 9.13	23.55 ± 12.24	20.02 ± 5.75	9.566	0.000
Prevalence of elevated ALT (%)	211 (6.8)	154 (12.2)	57 (3.1)	98.498	0.000
Prevalence of elevated AST (%)	71 (2.3)	52 (4.1)	19 (1.0)	32.116	0.000
Current smoking status (%)	76 (2.4)	68 (5.4)	8 (0.4)	77.192	0.000
Current drinking status (%)	428 (13.7)	329 (26.0)	99 (5.4)	270.812	0.000
Prevalence of hypertension (%)	262 (8.4)	203 (16.1)	59 (1.9)	161.438	0.000

BMI, body mass index; SBP, systolic blood pressure; DBP, diastolic blood pressure; GLU, glucose; TC, total cholesterol; TG, triglycerides; HDL-C, high-density lipoprotein cholesterol; LDL-C, low-density lipoprotein cholesterol; ALT, alanine aminotransferase; AST, aspartate aminotransferase

### Prevalence of hypertension

The overall prevalence of hypertension was 8.4% (n = 262). Participants with hypertension had higher BMI, TC, TG, LDL-C, ALT, and AST, and had lower HDL-C levels. The prevalence abnormal liver enzymes rate was significantly higher in the hypertensive group (ALT 21.0% vs. 5.5%; AST 6.5% vs. 1.9%; obesity 30.9% vs. 10.5%, *P* < 0.05) than in the normotensive group. In males, the mean ALT and AST concentration were higher in those with hypertension. In contrast, no differences were observed among the female participants (Table 2).

**Table 2:** Anthropometric and metabolic characteristics categorized by hypertension and sex

Characteristic	Total (n = 3114)		*t*/*χ*^2^	*P*	Males (n = 1264)		*t*/*χ*^2^	*P*	Females (n = 1850)		*t*/*χ*^2^	*P*
	Normotension (n = 2852)	Hypertension (n = 262)			Normotension (n = 1061)	Hypertension (n = 203)			Normotension (n = 1791)	Hypertension (n = 59)		
Age (year)	18.52 ± 1.01	18.42 ± 1.03	1.594	0.111	18.58 ± 1.01	18.51 ± 1.02	0.935	0.350	18.49 ± 1.01	18.12 ± 1.04	2.786	0.005
BMI (kg/m^2^)	22.09 ± 3.27	25.04 ± 4.90	− 9.575	0.000	22.68 ± 3.61	25.37 ± 4.91	− 7.444	0.000	21.73 ± 2.99	23.91 ± 4.75	− 3.492	0.001
GLU (mmol/L)	4.57 ± 0.40	4.55 ± 0.42	0.563	0.573	4.51 ± 0.38	4.54 ± 0.41	− 0.867	0.386	4.6 ± 0.41	4.62 ± 0.45	− 0.22	0.826
TC (mmol/L)	4.04 ± 0.67	4.17 ± 0.82	− 2.374	0.018	3.97 ± 0.71	4.2 ± 0.86	− 4.045	0.000	4.08 ± 0.63	4.05 ± 0.65	0.437	0.662
TG (mmol/L)	0.82 ± 0.38	0.98 ± 0.55	− 4.731	0.000	0.84 ± 0.45	1 ± 0.56	− 3.717	0.000	0.81 ± 0.34	0.93 ± 0.49	− 1.915	0.060
HDL-C (mmol/L)	1.43 ± 0.27`	1.27 ± 0.24	9.897	0.000	1.33 ± 0.25	1.24 ± 0.21	5.273	0.000	1.49 ± 0.27	1.39 ± 0.32	2.865	0.004
LDL-C (mmol/L)	2.07 ± 0.54	2.30 ± 0.72	− 5.000	0.000	2.11 ± 0.6	2.35 ± 0.75	− 5.017	0.000	2.05 ± 0.5	2.11 ± 0.55	− 0.979	0.328
ALT (U/L)	15.63 ± 14.89	27.96 ± 28.51	− 6.917	0.000	20.96 ± 19.64	31.10 ± 30.18	− 4.606	0.000	12.47 ± 9.90	17.15 ± 18.20	− 1.967	0.054
AST (U/L)	21.06 ± 7.99	25.74 ± 19.71	− 3.822	0.000	22.92 ± 9.26	26.86 ± 21.75	− 2.539	0.012	19.95 ± 5.60	21.90 ± 9.07	− 1.635	0.107
Prevalence of elevated ALT (%)	156 (5.5)	55 (21.0)	91.530	0.000	105 (9.9)	49 (24.1)	32.302	0.000	51 (2.8)	6 (10.2)	7.949	0.005
Prevalence of elevated AST (%)	54 (1.9)	17 (6.5)	22.741	0.000	37 (3.5)	15 (7.4)	6.577	0.010	17 (0.9)	2 (3.4)	1.377	0.241
Prevalence of obesity (%)	299 (10.5)	81 (30.9)	93.501	0.000	137 (12.9)	67 (33.0)	50.827	0.000	162 (9.0)	14 (23.7)	14.306	0.000
Current smoking status (%)	65 (2.3)	11 (4.2)	3.713	0.054	57 (5.4)	11 (5.4)	0.001	0.979	8 (0.4)	0 (0)	−	0.771
Current drinking status (%)	373 (13.1)	55 (21.0)	12.676	0.000	277 (26.1)	52 (25.6)	0.021	0.884	96 (5.4)	3 (5.1)	0.001	0.999

BMI, body mass index; SBP, systolic blood pressure; DBP, diastolic blood pressure; GLU, glucose; TC, total cholesterol; TG, triglycerides; HDL-C, high-density lipoprotein cholesterol; LDL-C, low-density lipoprotein cholesterol; ALT, alanine aminotransferase; AST, aspartate aminotransferase

### Levels of demographic and clinical variables in the serum ALT and AST quartiles

Baseline information of the subjects in each serum ALT and AST quartile is presented in Table 3. The mean values of BMI, SBP, DBP, TC, TG, and LDL-C were found to be increased with elevated levels of serum ALT and AST in the quartiles.

**Table 3:** Baseline characteristics of the study participants according to ALT and AST quartiles

Characteristic	ALT (U/L)				*P*-values for trend	AST (U/L)				*P*-values for trend
	Q1 (≤ 9)	Q2 (10–12)	Q3 (13–18)	Q4 (≥ 19)		Q1 (≤ 17)	Q2 (18–20)	Q3 (21–23)	Q4 (≥ 24)	
Age (year)	18.50 ± 1.05	18.49 ± 1.01	18.53 ± 0.98	18.56 ± 0.98	0.162	18.50 ± 1.04	18.54 ± 1.05	18.54 ± 0.98	18.48 ± 0.94	0.651
BMI (kg/m^2^)	20.89 ± 2.38	21.56 ± 2.86	22.29 ± 3.03	25.15 ± 4.21	0.000	21.68 ± 2.81	21.69 ± 2.96	22.22 ± 3.32	24.11 ± 4.5	0.000
SBP (mmHg)	110.01 ± 11.82	111.42 ± 12.6	115.97 ± 13.35	120.49 ± 14.89	0.000	112.39 ± 12.58	112.20 ± 13.08	114.41 ± 13.54	118.25 ± 15.13	0.000
DBP (mmHg)	69.18 ± 9.85	69.96 ± 10.22	71.06 ± 10.38	74.21 ± 11.24	0.000	70.27 ± 10.11	69.99 ± 10.19	70.91 ± 10.36	73.07 ± 11.44	0.000
GLU (mmol/L)	4.56 ± 0.40	4.57 ± 0.38	4.56 ± 0.39	4.58 ± 0.44	0.560	4.58 ± 0.41	4.57 ± 0.40	4.57 ± 0.38	4.54 ± 0.42	0.077
TC (mmol/L)	3.97 ± 0.60	4.02 ± 0.65	4.00 ± 0.66	4.25 ± 0.79	0.000	3.96 ± 0.60	4.00 ± 0.65	4.08 ± 0.69	4.21 ± 0.78	0.000
TG (mmol/L)	0.75 ± 0.26	0.78 ± 0.34	0.82 ± 0.39	1.01 ± 0.55	0.000	0.77 ± 0.28	0.80 ± 0.35	0.85 ± 0.43	0.95 ± 0.53	0.000
HDL-C (mmol/L)	1.47 ± 0.25	1.47 ± 0.27	1.40 ± 0.28	1.30 ± 0.28	0.000	1.44 ± 0.26	1.43 ± 0.27	1.43 ± 0.27	1.36 ± 0.29	0.000
LDL-C (mmol/L)	1.98 ± 0.45	2.00 ± 0.50	2.06 ± 0.53	2.36 ± 0.66	0.000	2.01 ± 0.47	2.03 ± 0.51	2.10 ± 0.56	2.27 ± 0.66	0.000

BMI, body mass index; SBP, systolic blood pressure; DBP, diastolic blood pressure; GLU, glucose; TC, total cholesterol; TG, triglycerides; HDL-C, high-density lipoprotein cholesterol; LDL-C, low-density lipoprotein cholesterol; ALT, alanine aminotransferase; AST, aspartate aminotransferase

(*P* < 0.05 for trend).

### Correlation between ALT and AST levels and hypertensive risk factors

Serum ALT levels were positively correlated with BMI, SBP and DBP in both genders (*r* = 0.511, 0.207, 0.172, respectively; *P* < 0.05). Serum AST levels were positively correlated with BMI, SBP and DBP in males, but only with BMI and DBP in females (*P* < 0.05) (Table 4).

**Table 4:** Correlation between ALT and AST levels and hypertensive risk factors

Characteristic	ALT						AST					
	Total		Males		Females		Total		Males		Females	
	*r*	*P*	*r*	*P*	*r*	*P*	*r*	*P*	*r*	*P*	*r*	*P*
Age	0.018	0.318	0.010	0.722	0.000	0.999	− 0.008	0.661	− 0.023	0.420	− 0.010	0.665
BMI	0.451	0.000	0.511	0.000	0.295	0.000	0.264	0.000	0.291	0.000	0.156	0.000
SBP	0.273	0.000	0.206	0.000	0.105	0.000	0.150	0.000	0.090	0.001	0.045	0.051
DBP	0.177	0.000	0.172	0.000	0.088	0.000	0.093	0.000	0.069	0.014	0.058	0.013
GLU	0.018	0.319	0.047	0.091	0.070	0.003	− 0.007	0.679	0.006	0.829	0.027	0.246
TC	0.153	0.000	0.252	0.000	0.066	0.005	0.104	0.000	0.151	0.000	0.070	0.003
TG	0.266	0.000	0.310	0.000	0.171	0.000	0.130	0.000	0.136	0.000	0.094	0.000
HDL-C	− 0.223	0.000	− 0.213	0.000	− 0.085	0.000	− 0.120	0.000	− 0.119	0.000	− 0.006	0.804
LDL-C	0.258	0.000	0.311	0.000	0.139	0.000	0.156	0.000	0.177	0.000	0.089	0.000

BMI, body mass index; SBP, systolic blood pressure; DBP, diastolic blood pressure; GLU, glucose; TC, total cholesterol; TG, triglycerides; HDL-C, high-density lipoprotein cholesterol; LDL-C, low-density lipoprotein cholesterol; ALT, alanine aminotransferase; AST, aspartate aminotransferase

### Association of ALT and AST with hypertension

After adjusting for age and sex, the odd ratios (ORs) (95% CI) were 1.774 (1.139, 2.762), and 3.047 (2.028, 4.578), respectively in Q3 and Q4 compared to Q1 of the ALT. After additionally adjusting obesity, smoking, and drinking, TC, TG, LDL-C, HDL-C, compared with those in the lowest quartile, individuals in the highest quartile of serum ALT levels had associated with hypertension in males and females, with an OR (95% CI) of 1.681 (1.028, 2.751), and 2.802 (1.102, 7.124), respectively. However, there was no significant association of serum AST levels with hypertension in the full adjustment model (Table 5).

**Table 5:** Association of ALT and AST with hypertension

Group	Characteristic	Model 1	Model 2	Model 3
Total	ALT			
	Q1 (≤ 9)	1	1	1
	Q2 (10–12)	1.575 (0.992, 2.500)	1.517 (0.955, 2.409)	1.511 (0.950, 2.402)
	Q3 (13–18)	1.774 (1.139, 2.762)*	1.614 (1.033, 2.523)*	1.545 (0.986, 2.420)
	Q4 (≥ 19)	3.047 (2.028, 4.578)*	2.111 (1.365, 3.266)*	1.815 (1.157, 2.846)*
	*P* for trend	0.000	0.001	0.014
	AST			
	Q1 (≤ 17)	1	1	1
	Q2 (18–20)	1.446 (0.964, 2.167)	1.471 (0.979, 2.212)	1.435 (0.953, 2.162)
	Q3 (21–23)	1.479 (0.966, 2.264)	1.354 (0.879, 2.085)	1.329 (0.860, 2.054)
	Q4 (≥ 24)	2.000 (1.355, 2.953)*	1.502 (1.001, 2.254)*	1.388 (0.917, 2.099)
	*P* for trend	0.001	0.102	0.219
Males	ALT			
	Q1 (≤ 11)	1	1	1
	Q2 (12–16)	1.409 (0.884, 2.243)	1.319 (0.826, 2.107)	1.245 (0.776, 1.966)
	Q3 (17–25)	1.401 (0.874, 2.245)	1.166 (0.718, 1.893)	1.031 (0.630, 1.688)
	Q4 (≥ 26)	3.139 (2.076, 4.746)*	2.033 (1.274, 3.245)*	1.681 (1.028, 2.751)*
	*P* for trend	0.000	0.006	0.076
	AST			
	Q1 (≤ 18)	1	1	1
	Q2 (19–21)	1.152 (0.731, 1.814)	1.121 (0.708, 1.776)	1.098 (0.691, 1.746)
	Q3 (22–25)	1.533 (0.991, 2.371)	1.33 (0.852, 2.077)	1.275 (0.81, 2.009)
	Q4 (≥ 26)	1.88 (1.233, 2.868)*	1.276 (0.812, 2.007)	1.151 (0.725, 1.828)
	*P* for trend	0.001	0.224	0.462
Females	ALT			
	Q1 (≤ 7)	1	1	1
	Q2 (8–9)	1.442 (0.508, 4.094)	1.421 (0.499, 4.040)	1.430 (0.502, 4.071)
	Q3 (10–13)	2.660 (1.062, 6.662)*	2.453 (0.976, 6.170)	2.516 (0.996, 6.356)
	Q4 (≥ 14)	3.444 (1.386, 8.557)*	2.916 (1.157, 7.351)*	2.802 (1.102, 7.124)*
	*P* for trend	0.002	0.008	0.012
	AST			
	Q1 (≤ 16)	1	1	1
	Q2 (17–19)	1.721 (0.724, 4.093)	1.794 (0.751, 4.283)	1.784 (0.747, 4.263)
	Q3 (20–21)	1.398 (0.514, 3.802)	1.409 (0.516, 3.846)	1.409 (0.514, 3.865)
	Q4 (≥ 22)	2.554 (1.077, 6.055)*	2.395 (1.005, 5.711)*	2.398 (0.998, 5.763)
	*P* for trend	0.042	0.076	0.078

Model 1: Adjusted for age, gender

Model 2: Adjusted for model 1 plus, obesity (based on BMI), smoking and drinking

Model 3: Adjusted for model 2 plus, TC, TG, HDL, LDL

*Compared to Q1, *P* < 0.05

## Discussion

In this study, we evaluated the relationship between ALT and AST levels and hypertension in a large, representative sample of 3114 freshmen. We demonstrated that only serum ALT, but not AST, was associated with hypertension both in males and females after adjusting for potential confounders.

In recent years, the incidence of abnormal ALT and AST has increased worldwide, making it a health issue [18, 19]. The overall prevalence of elevated ALT and AST were 6.8% and 2.3% in our study. A school-based cross-sectional study in China reported a prevalence of 6.76% for ALT levels greater than 30 U/L for boys and 9 U/L for girls, compared with 2.43% at a cut-off level of greater than 40 U/L [20]. Asymptomatic elevation in ALT levels is common in primary care, and the prevalences of elevated ALT and AST in the United States were 8.9%, 4.9% respectively [21]. Freshmen are a special group and some biomarker levels are quite different from those of healthy adults [22]. This study shows that the prevalence of elevated ALT and AST levels differed in gender among freshman of China. Male freshmen had a significantly higher prevalence of elevated ALT and AST than female freshmen. We should take the prevalence as a predictor and continue to play a warning and preventive role.

ALT refers to an enzyme that is largely found in the liver. When the liver is damaged, ALT will be sent to the bloodstream, signalling liver disease, and ALT level in blood is used as an indication of the liver status, giving information about the seriousness of liver damage. Although AST is a marker of hepatocellular health, it is a less specific marker of liver function than ALT [23]. Our results revealed the SBP and DBP were increasing along with serum ALT and AST elevating whether in females or males populations. However, in regression analysis, only ALT was significantly associated with hypertension even after adjustment of potential confounders. A similar finding was observed in a previous study that only serum ALT showed an independent relationship with hypertension [24]. More and more studies have shown that ALT play a considerable role in the development of cardiovascular diseases [18, 25]. Besides, ALT is associated with endothelial dysfunction and coronary heart disease [26]. A Korea Medical Insurance Corporation study showed that an elevated ALT is a predictor of intracerebral hemorrhage [27]. A longitudinal increase in ALT activity increases the incidence of metabolic syndrome [28]. Elevated plasma ALT levels were associated with hypertension at baseline in the Hong Kong Cardiovascular Risk Factor Prevalence Study. For participants who were not on antihypertensive medications, the plasma ALT level correlated with high blood pressure [29].

Although the mechanism underlying the associations between ALT and hypertension remains unclear, some mechanisms can be considered, and one of the most plausible is non-alcoholic fatty liver disease (NAFLD). Elevated serum ALT levels reflect an excess deposit of fat in the liver and are commonly used as a surrogate marker for NAFLD in epidemiological studies [30]. NAFLD represents the hepatic manifestation of the metabolic syndrome, which refers to a variably defined aggregate of disorders related to obesity, hypertension, type 2 diabetes, and hyperlipidemia [31]. Lopez-Suarez A et al. [32] indicated that NAFLD is associated with an independent risk of identifying hypertension and high-normal SBP. Detection of NAFLD, even with normal ALT levels, should serve as an opportunity to identify metabolic and BP abnormalities. Overweight and obesity are associated with elevated biochemical markers of liver damage, and obesity is well-known major predisposing conditions to NAFLD [33]. Children and adolescents who are overweight or obese have shown higher ALT concentrations in all age groups [34]. Our results also showed that ALT had the strongest correlation with BMI than other hypertension risk factors. A study of Poland middle and high school students aged 15–17 found that BMI is one of the strongest predictors of hypertension in teenagers [35]. A previous study revealed that an increase in BMI is linked to hypertension in university students [36]. Obesity may lead to hypertension in multiple ways by different percents [37]. It is speculated that high ALT levels will lead to an increase in BMI, which in turn leads to hypertension. Other possibility is that elevated serum ALT is a marker of inflammation and oxidative stress, and oxidative stress may play an important role in the initiation and progression of hypertension [38]. Finally, insulin resistance is the most common abnormality associated with the pathogenesis of both ALT and hypertension. ALT is associated with insulin resistance [39], and insulin resistance is commonly detected in individuals with hypertension [40].

We present the gender-specific analysis for the association between increasing liver enzymes levels and hypertension. In our logistic regression analysis, after adjusting for hypertensive risk factors, the risk of hypertension was significantly higher in the highest ALT quartile than in the lowest quartile in both sexes. However, a clear linear association between ALT and hypertension was present only among females. We did not observe an association between elevated serum AST levels and hypertension. One reason for the lack of linear association between ALT and hypertension in males may be related to sex differences in the absolute risk of hypertension. Research has reported that the gender difference of blood pressure began to appear in adolescence, and pubertal growth spurt occurs earlier for females than for males [41]. Thus, the relationship between ALT levels and hypertension in males needs to be further investigated.

The limitations of this study are as follows. First, this was a cross-sectional study, and causality could not be determined. Thus, further prospective studies are needed to verify the causal relationship between ALT and the prevalence of hypertension. Second, the study participants were all freshmen, so the results may not be generalizable to students of other age groups.

## Conclusions

Results of this study suggest that serum ALT but not AST levels were positively associated with the prevalence of hypertension in both sexes. A linear association between elevated serum ALT and hypertension only in total population and females. To understand the complex interaction between ALT and hypertension, a large, well-constructed prospective study to elucidate the association between these factors is indicated.

## Data Availability

The datasets during and/or analyzed during the current study will be available from the corresponding author on reasonable request.

## Abbreviations

ALT: Alanine aminotransferase
AST: Aspartate aminotransferase
BMI: Body mass index
BP: Blood pressure
SBP: Systolic blood pressure
DBP: Diastolic blood pressure
GLU: Glucose
TC: Total cholesterol
TG: Triglycerides
HDL-C: High-density lipoprotein cholesterol
LDL-C: Low-density lipoprotein cholesterol
UA: Uric acid
